# Heterogeneity of dementia with Lewy bodies: Insights from clinical presentations, neuropathology, and biomarkers

**DOI:** 10.1007/s10072-025-08725-3

**Published:** 2025-12-23

**Authors:** Beatrice Orso, Ariane Bollack, Zulfiqar H. Sheikh, Silvia Morbelli, Matteo Pardini, Gill Farrar

**Affiliations:** 1 Department of Neuroscience, Genetics, Maternal and Child Health (DINOGMI), Rehabilitation, Ophthalmology, University of Genoa, Largo Daneo 3, 16132 Genoa, Italy; 2 GE HealthCare, Pharmaceutical Diagnostics, Nightingales Ln, Chalfont Saint Giles, HP8 4SP UK; 3 Centre for Medical Image Computing (CMIC), Department of Medical Physics and Bioengineering, University College London, London, UK; 4 Nuclear Medicine Unit, AOU Città Della Salute E Della Scienza Di Torino, Torino, Italy; 5 Department of Medical Sciences, University of Turin, Turin, Italy; 6 Clinical Neurology Unit, IRCCS Ospedale Policlinico S. Martino, Genoa, Italy

**Keywords:** Dementia with Lewy Bodies, Heterogeneity, Biomarkers, Disease progression

## Abstract

**Background:**

Dementia with Lewy Bodies (DLB) is a neurodegenerative disorder characterised by α-synuclein pathology, causing cognitive decline, motor and non-motor symptoms. This review explores the clinical and neuropathological heterogeneity of DLB, which complicates its early diagnosis, prognosis, and treatment. The staging of Lewy body (LB) pathology varies, with both brain-first and body-first hypotheses suggesting different origins and pathways for disease progression. Co-pathologies, such as amyloid-β plaques, tau tangles, and cerebrovascular changes, further influence the clinical presentation and rate of disease progression in DLB patients, contributing to significant variability. In this review, the role of genetic factors, particularly APOE ε4 and GBA mutations, in shaping DLB’s clinical and pathological diversity is also emphasized. Heterogeneous manifestations, including REM sleep behavior disorder (RBD), mild cognitive impairment, and psychiatric-onset DLB, highlight the need for improved biomarkers to guide early diagnosis. Neuroimaging techniques such as [^18^F]FDG PET and [^123^I]FP-CIT SPECT help distinguish DLB from other dementias, like Alzheimer’s disease (AD), though challenges remain in identifying co-pathologies with precision.

**Conclusion:**

Overall, the paper explores the complexity of DLB’s heterogeneous nature, advocating for deeper exploration of its diverse pathological pathways, genetic predispositions, and clinical profiles to improve diagnosis and treatment outcomes. Understanding this heterogeneity is crucial for the development of personalized therapeutic strategies and effective management of the disease.

## Introduction

Dementia with Lewy Bodies (DLB) is an age-associated neurodegenerative disorder that leads to progressive cognitive decline, interfering with normal life and daily activities[1], and represents one of the most common cause of dementia [2].

The disease is characterized by the accumulation of abnormal α-synuclein aggregates, known as Lewy bodies (LBs), in neuronal cell bodies as well as neurites in neuronal processes [3]. Dementing conditions associated with Lewy bodies pathology are generally identified as DLB and Parkinson’s Disease Dementia (PDD). Both PDD and DLB are associated with relatively wide phenotypical heterogeneity in terms of prevalent clinical features and prognosis. However, the definition of PDD is more straightforward, manifesting at first with parkinsonism, followed by cognitive decline more than one year after[1, 4], while DLB exhibits remarkably greater heterogeneity, with regards to clinical presentation, symptoms onset and progression, partially related to a higher variety of underlying pathological features. However, this heterogeneity extends beyond its neuropathological characteristics and contributes to the complexity of the disease, impacting both clinical presentation and progression. Indeed, a recent meta-analysis found that 20% of DLB diagnoses were incorrect, with AD being the most frequent misdiagnosis [5].

To date, a major shift from a clinical definition to a biological one has been introduced in the research of Lewy Body diseases, also known as α-synucleinopathies representing an effort to overcome the heterogeneity of clinical syndromes and clinical progression among these conditions. Simuni et al. (2024)[6] suggested that both PD and DLB might be classified as Neuronal α-synuclein Diseases (NSD), highlighting the possibility of redefining these syndromes based on biology rather than clinical features. This has become possible due to advances of new assays, which accurately allow in-vivo assessment of neuronal α-synuclein (n-αsyn), using cerebrospinal fluid (CSF). The first biological anchor to define NSD is the presence of pathological n-αsyn (S) regardless of the presence of any clinical syndromes, if pathological n-αsyn is detected (S +), it represents a higher risk of having dopaminergic dysfunction (D), which represents the second biological anchor [6]. This has led to defining a staging system linked to the new biological classification of NSD, namely the neuronal a-synuclein disease integrated staging system (NSD-ISS), which aims to establish a research framework for therapeutic development, starting from the initial stages, when pathological changes are detectable but there are no clinical symptoms or functional impairments, and continuing through to advanced disease [6]. This transition to the NSD-ISS redefines the approach to these neurodegenerative conditions, but also underscores the critical role of diagnostic and progression biomarkers for DLB, facilitating earlier and more targeted interventions.

In this perspective review, we aim to delve into current state-of-the-art developments regarding the multifaceted nature of DLB, shedding light on challenges posed by its heterogeneity, following a narrative review approach. A central component of this article is the value that biomarkers can bring as researchers and clinicians seek to disentangle the intricate tapestry of this disorder.

To ensure a comprehensive review of the literature, we conducted a detailed search using the following databases: PubMed, Embase, and Scopus. The search strategy included keywords such as "Dementia with Lewy Bodies," "heterogeneity," "biomarkers," "genetics," and "disease progression." Studies were selected based on their relevance to the topic, with inclusion criteria focusing on peer-reviewed articles published in English, involving human subjects, and providing original data or comprehensive reviews. Quality assessment was performed to ensure the inclusion of high-quality studies.

## Neuropathologic heterogeneity and Genetic influence

### α-synuclein pathology: differences in initiation and propagation

**Fig. 1:**
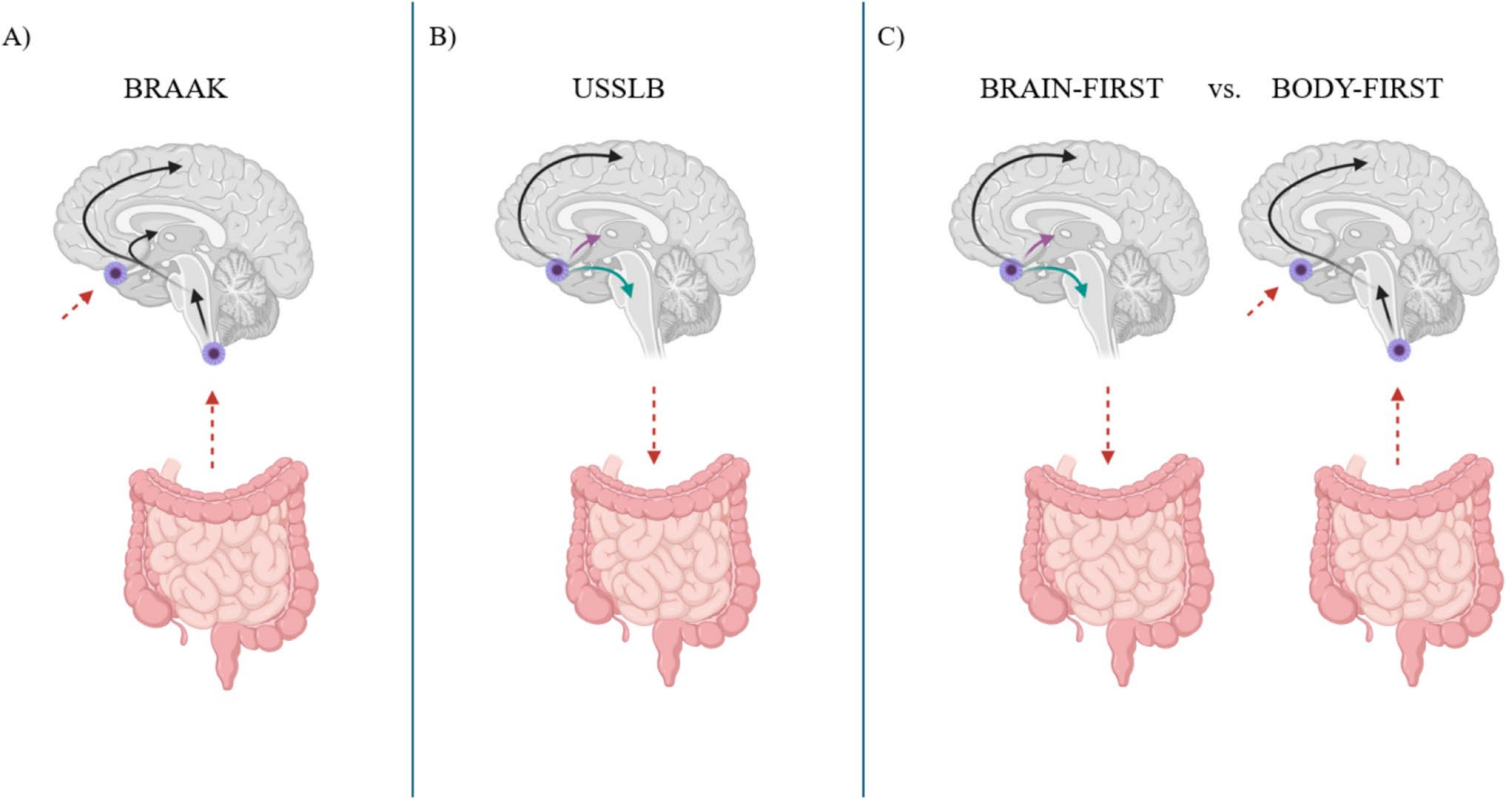
Representation of three models of Lewy bodies-related pathology progression. **A)** The Braak staging model. This model illustrates the deposition of Lewy bodies (LBs) starting from peripheral regions and progressing to either the brainstem and/or the olfactory bulb. **B)** The Unified Staging System for Lewy Body disorders (USSLB). This model depicts LBs pathology initiating in the brain and spreading to either the brainstem (indicated by a green arrow) or limbic regions (indicated by a purple arrow). **C)** The brain-first vs. body-first model. This model hypothesizes the existence of two subtypes of LB pathology, where LBs can originate either in the brain or in peripheral organs, such as the intestines. *Figure created using Biorender*

Over the years, researchers have focused on the definition of an accurate staging system that clearly describes the pathological progression of LBs. One of the most well-known staging schemes is the Braak staging scheme[7], where LB disease may have peripheral origins and enter the brain by intestinal and olfactory epithelium, starting concurrently in lower brainstem areas and the olfactory bulb [7]. Brainstem Lewy body pathology is generally thought to progress in a caudal-to-rostral manner, beginning in the medulla and ascending to the limbic regions and ultimately the neocortex. (Fig. 1A). Alternatively, the Unified Staging System for Lewy Body disorders (USSLB)[8], asserts that LB pathology typically starts in the olfactory bulb, then splits off into a limbic- or brainstem-predominant pathway and eventually spreads to cortical areas (Fig. 1B). The brain-first and body-first hypothesis acknowledges the existence of two subtypes of LB staging. The brain-first describes LB pathology as originating from the olfactory regions and subsequently follows either limbic or brainstem pathways; while the body-first identifies LB pathology as starting from the gut and expanding through brainstem regions (Fig. 1C) [9].

Multiple diverse spatiotemporal subtypes of LB staging have been described [8, 10]. Mastenbroek et al*.* (2024), in a 814 subjects cohort, identified early olfactory bulb pathology, extending to limbic or brainstem regions, in 82% of individuals. In the remaining subjects, early abnormalities were observed in brainstem regions. A slower clinical decline at follow-up was associated with deposition of LBs in the olfactory bulb and brainstem, while the early deposition of LBs in the limbic regions was associated with AD-like characteristics (APOE ε4 carriers, AD pathology and cognitive functioning)[10], which highlight that the diverse involvement of brain regions, along with associated AD risk factors, might results in heterogeneous DLB presentation.

Understanding the presence of co-pathologies guides clinical and research efforts by highlighting the need to investigate specific interactions between different pathological processes. As co-pathologies also influence the rate of disease progression and overall prognosis in DLB and its phenotypes, this knowledge could contribute to the development of more specific targeted therapies and potential disease-modifying interventions.

### Amyloid-β (Aβ) plaques and Tau neurofibrillary tangles

Amyloid-β and Tau accumulation are central to AD pathology, and can also be found in more than 50% of DLB patients [2, 11]. Presence of these AD-like features in Lewy Body Disorders (LBD) has been linked to faster cognitive decline, a shorter interval between motor symptoms onset and dementia, as well as to a shorter survival time [12].

The molecular relationship between LB and AD-type pathology is still unclear. One possibility is that amyloid-β deposition serves as the initial event in both AD and DLB, with the subsequent effects being either tau aggregation, leading to an AD-like clinical presentation, or α-synuclein aggregation, leading to a DLB-like presentation. However, this hypothesis is not universally supported, as there are DLB cases lacking Aβ pathology. In some patients, the coexistence of these overlapping pathologies produces a mixed or unclear clinical profile [13].

Recent reviews by Barba et al. (2024)[14] and Toledo et al. (2023)[11] have emphasized that these pathologies may not develop independently, but rather influence each other’s accumulation and spatial distribution. Several models have been proposed to explain their relationship:

- Synergistic process: some evidence suggests that Aβ deposition facilitates both tau phosphorylation and α-synuclein aggregation, possibly by impairing proteostasis or through overlapping pathways of neuroinflammation [15]. In turn, tau and α-synuclein may act synergistically to exacerbate neurodegeneration. For instance, digital histological studies have shown that tau and Aβ burden correlates with higher cortical α-synuclein pathology [16].
- Sequential process: another view posits that Aβ deposition is the initiating event, followed either by tau aggregation (leading to an AD-like trajectory) or α-synucleinopathy (leading to a DLB trajectory), with some patients developing both pathways [17, 18]. The directionality and timing of these changes may determine the predominant clinical phenotype.
- Independent or Parallel processes: alternatively, it has been argued that Aβ, tau, and α-synuclein may accumulate independently due to aging or genetic predisposition, with co-pathologies converging in vulnerable brain regions. [19] Ô This could explain the significant phenotypic heterogeneity even among patients with similar pathological burdens.

Neuroimaging studies with Amyloid PET studies showed that DLB is associated with lower mean cortical Aβ ligand binding than AD [20]. Although, when Aβ accumulation is found in DLB, in form of diffuse rather than neuritic plaques, the consequent deposition is similar to the one found in AD patients, with distribution in frontal, parietal, and cingulate areas, along with the striatum [20].

Walker et al. (2015) showed a clear difference in the distribution of hyperphosphorylated tau load between clinical AD and clinical DLB patients, with the former showing a higher burden in temporal cortex, while the latter in the occipital cortex [21].

More recently, CSF levels of pathological total tau and phosphorylated tau have been detected in almost 28% and 25% of DLB patients, respectively [22]. Evidence of a high tau burden in some of the DLB patients might hinder early clinical diagnosis, since these patients are less likely to manifest the core clinical features, such as visual hallucination and extrapyramidal signs.[23].

In summary, the interaction between Aβ, tau, and α-synuclein pathologies is multifaceted. Exploring whether these pathologies act sequentially, synergistically, or in parallel is crucial to better understand DLB heterogeneity. Recent integrative frameworks, such as that proposed by Barba et al. (2024)[14], advocate for pathology-driven phenotyping to improve diagnostic accuracy and guide targeted therapeutic development.

### Genetic underpinnings

It is hypothesized that certain genes and loci linked to DLB, as well as the degree of linkage, contribute to the heterogeneity seen in clinical and pathological presentation of this condition.

*SNCA* point mutations and gene dosage are the only fully penetrant genetic alterations that have been found and confirmed as a particular cause of DLB thus far [24]. Nonetheless, it is currently undeniable that DLB has a significant genetic component. Strong risk factors include the APOE ε4 allele, heterozygous mutations, and frequent polymorphisms in the glucocerebrosidase gene (*GBA*) [24].

Guerreiro et al. (2018)[24], conducted a study on 1216 DLB patients, 974 of which were neuropathologically assessed, and included 3791 controls. They identified a genome-wise association at the *APOE* locus, which has also been reported to affect the levels of both amyloid-β and LB pathology, *SNCA* and *GBA*, loci that have also been implicated in PD and AD.

Recently, Kaivola et al. (2022)[25], using a an APOE-stratified genome-wide association study approach on 2466 DLB patients and 2928 healthy controls, explored whether distinct genetic architectures influence different DLB phenotypes. They found that *GBA* associated with risk for DLB in those patients without APOE ε4, but not with those with APOE ε4. To further study the possible role of co-pathology, the authors divided their sample of 495 neuropathologically confirmed DLB into three groups, based on the extent of AD co-pathology, as no AD co-pathology (pure DLB; n = 88), intermediate (n = 66) and high (n = 341) AD co-pathology, and they tested the association of the APOE ε4 against the control group. They report that APOE ε4 was associated with DLB patients with both intermediate and high AD co-pathology, but not with pure DLB cases, concluding that APOE ε4 does not represent an independent driver of a-synuclein pathology but, on the other hand, its association with DLB is dependent on the severity of AD co-pathology [25].

## Heterogeneity of clinical manifestations

### REM sleep behaviour disorder

REM sleep behavior disorder (RBD) is a parasomnia characterized by dream-enacting behaviours that emerge during REM sleep given by the loss of physiological muscle atonia [26]. A clinical diagnosis of RBD is carried out in presence of REM sleep without atonia (RWA) recorded by an overnight video-polysomnography (vPSG), which shows repetitive episodes of vocalizations and/or complex motor behaviours occurring during REM sleep [27].

RBD represents a specific manifestation of the prodromal stage of α-synucleinopathies. During follow-up, up to 70% individuals with isolated/idiopathic RBD (iRBD) eventually develop parkinsonism and/or dementia within 12 years from diagnosis [28].

Back in 1998, Boeve et al.[29] found that 34 out of 37 iRBD patients met the criteria for possible/probable DLB, paving the way to the hypothesis of DLB being the underlying pathology in iRBD subjects. Indeed, DLB patients with iRBD present more severe parkinsonian symptoms, autonomic dysfunctions and cognitive impairment when phenoconverting, compared to those without evidence of iRBD at baseline [30].

In DLB patients with iRBD at baseline, neuroimaging studies showed a lower burden of phospho-tau and Aβ in both hippocampus and amygdala, compared to those without iRBD, who instead showed a greater atrophy in temporoparietal cortices, hippocampus and amygdala; concluding that having RBD at baseline was associated with a higher likelihood of developing DLB as an overt stage of α-synucleinopathy, and showing a less severe AD-related pathology [31].

From a neuropsychological standpoint, it has been shown that those iRBD patients converting to DLB within 6-years follow-up, present with worse attention and executive function already at baseline, suggesting that, the use of an executive function test, such as the TMT part B, might be useful for identifying iRBD patients at risk of dementia and maybe valuable for clinical trial inclusion [32].

### Mild cognitive impairment

Mild cognitive impairment (MCI) can occur in the early stages of DLB and is often considered a transitional stage between normal aging and dementia [33]. It is characterized by complaints in one (single-domain) or more (multiple-domain) cognitive domains, reported by either patient or caregiver, and objectively assessed through an extensive neuropsychological evaluation, with minimal-to-none interference with activities of daily living. [34]

In patients with MCI, presence of RBD and parkinsonism at early stages are strongly associated with phenoconversion to DLB rather that AD or other dementing conditions[35].

Different clinical phenotypes of MCI-LB have been described. In a recent 34 MCI-LB patient study, Chen et al. (2021)[36] identified four different phenotypes based on amyloid-β PET and DATSCAN positivity (A +, A-; D +, D-; respectively). The largest group was represented by the A-D + phenotype (38.2%), followed by A + D + and A-D-, both at 26.5%. The smallest group was the A + D-, which included 8.8% of the subjects. Overall, the A + groups were older, had lower MMSE and a higher frequency of APOE ε4 carriers, while the D + groups were more likely to have RBD [36].

Rossi et al. (2021)[37] showed that CSF α-syn RT-QuIC assay identified MCI-LB patients from cognitively unimpaired subjects with 95% sensitivity, 97% specificity, and had high specificity in neuropathologic controls, suggesting that positive CSF α-syn RT-QuIC could be considered a robust biomarker for prodromal DLB[37], as well as the prospective identification of probable MCI-LB [38].

From a neuropsychological point of view, the Trail Making Test (Part A), Boston Naming Test, Auditory Verbal Learning Test (retention), and the copy of the Rey-Osterrieth Complex Figure were shown to differentiate between DLB and AD with a sensitivity of 83% and a specificity of ~ 92%, in a cohort of 87 DLB, 138 AD patients and 103 normal controls [39]. More recently, it has been shown that tests of processing speed and alternative attention (i.e., Stroop test, Trail Making Test and phonemic fluency) successfully differentiate DLB from AD, as well from normal aging, and are also able to predict conversion from MCI to DLB [1].

Early identification and diagnosis of MCI in DLB patients is therefore essential for implementing appropriate pharmacological and non-pharmacological interventions to help maintain independence and quality of life.

### Delirium- and psychiatric- onset

Delirium and psychiatric symptoms can be prominent features of DLB and may precede cognitive decline.

Delirium per se is an acute-onset brain disorder, often associated with a concurrent medical condition. Presentation is heterogeneous, with core features such as inattentiveness, alteration of arousal and fluctuation in cognitive performance, consciousness, disturbances of sleep–wake cycle and daily functioning [40, 41].

DLB shares these features with delirium, making the diagnosis challenging, especially in the prodromal stages [42]. There are no current guidelines nor validated tools to diagnose delirium in DLB or, more extensively, within the LBD spectrum; but researchers can rely on specific criteria, namely DSM-V or ICD-10 [42].

Table 1 summarized similarities between DLB and Delirium.

**Table 1:** Similar features between Dementia with Lewy Bodies and Delirium. Adapted from Gore et al. (2015)[80]

Dementia with Lewy Bodies	Delirium	Similarities
Global cognitive impairment	Partial cognitive impairment	Yes
Cognitive fluctuation	Cognitive fluctuation	Yes
Visual hallucination	Visual hallucination	Yes
Parkinsonian signs	Motor disturbances	Some
Frequent falls	Risk of falls	Some
Alteration of arousal	Alteration of arousal	Yes
REM sleep behaviour disorder	Sleep–wake disturbances	Some
Emotional dysfunction	Emotional dysfunction	Yes

Other than the delirium-onset, DLB patients may also present with prominent late-onset psychiatric disorders [40].

A recent review by Gunawardana et al. (2024) focused on studies describing patients satisfying the proposed psychiatric-onset criteria of DLB, to better understand the risk of conversion towards dementia [43]. The most common psychiatric symptom is depression, which is highly reported both at onset (88%) and in the prodromal stage of DLB (100%) [43]. Female patients most commonly presented with early-onset psychiatric symptoms (63%), while male patients typically present with RBD [44].

Psychiatric- and delirium-onset variants are non-amnestic, non-motor portals of entry into the DLB disease continuum. Though frequently under-recognized, such onset variants have particular importance for early diagnostic specificity and patient phenotyping. Future prodromal DLB diagnostic framework must formally address these unusual presentations to help inform earlier diagnosis and safer, more tailored treatment approaches.

## Biomarkers

As detailed above, the complexity of DLB is characterized by significant heterogeneity in its neuropathology, genetics, and clinical presentations. Biomarkers play a crucial role in unravelling this heterogeneity, providing insights that can guide diagnosis, prognosis, and help develop new therapies. These biomarkers include imaging techniques and fluid biomarkers, each contributing uniquely to the understanding of DLB.

### Imaging biomarkers

Imaging biomarkers provide structural and molecular and metabolic insights into DLB, crucial for its differential diagnosis, especially at early stages.

#### Structural imaging

Structural imaging techniques, such as computerized tomography or magnetic resonance imaging (MRI) allows to identify brain atrophy patterns, white matter changes and cerebrovascular disease, features that are usually unique to specific conditions [45]. For example, a MRI hallmark of AD is usually medial temporal lobe atrophy with a sensitivity of 90% and a specificity of 84%, while DLB atrophy pattern usually involves posterior cortices and a spare of the hippocampus area with 78.7% sensitivity and almost 99% specificity [46]. The main advantages of this method includes: its wide availability and allowing a differential diagnosis between AD and DLB. On the other hand, limitations might include: its low specificity for α-synucleinopathy and the lack of direct assessment of protein aggregation.

#### Functional imaging

In addition to brain atrophy patterns, molecular imaging such as positron emission tomography (PET) with ^18^F-Fluorodeoxyglucose ([^18^F]FDG) enables the identification of brain glucose metabolic patterns [28, 47].

In DLB, the characteristic pattern highlighted by [^18^F]FDG PET is the "cingulate island sign", which refers to the relative preservation of posterior or mid-cingulate metabolism, and has been found to have specific prognostic significance [48]. In DLB patients with evidence of AD co-pathology, the island sign is much less prominent [49]. Hypometabolism in the occipital cortex is able to distinguish DLB from AD patients with 90% sensitivity and 80% specificity [50].

The degeneration of the nigrostriatal pathway assessed using the ^123^iodine FP-CIT SPECT ([^123^I]FP-CIT SPECT or DAT SPECT) is a well-established *in-vivo* biomarker for the differentiation of DLB from AD[51], with high accuracy of 86% (sensitivity 80%, specificity 92%) [52]. In DLB, a 40%−70% loss of striatal dopamine of is observed [53]. The overall uptake of the tracer is lower in DLB compared to AD patients, especially in the caudate nucleus and the anterior and posterior putamen [54]. However, in approximately 10% of DLB patients DAT imaging was found to be normal, especially in those DLB patients with AD-mixed pathology [55]. The utility and challenges of implementing DAT SPECT in the USA were recently described by O’Shea et al. (2024)[56]; whilst the visual inspection methodology has relatively high accuracy to differentiate DLB from AD and other dementia’s, the added value of quantitative software to supplement the qualitative visual read was noted [56]. Indeed, qualitative visual interpretations and the selection of regions of interest (ROIs) are susceptible to both inter-rater and intra-rater variability. To overcome this bias, DaTQUANT (GE Healthcare), one of several automated semi-quantification software programs for [^123^I]FP-CIT SPECT[57], works by applying predefined volume of interest (VOI), and it’s discrimination power across LBD and non-LBD conditions has been proven to be comparable with other semiquantitative methods [58]. When coupled with different imaging modalities (MRI, [^11^C]PiB and [^18^F]FDG PET), [^123^I]FP-CIT SPECT power of discrimination between DLB and AD patients ranged from 0.987 to 0.996 [59]. These results highlight the importance of utilize multimodal imaging biomarkers not only for recruiting reliable participants with DLB, but also for evaluating AD co-pathology, which will aid in stratifying patients in future clinical trials on DLB, involving disease-modifying therapies.

The main advantages of these methods include: the high diagnostic accuracy when distinguishing DLB from AD and their potential of supporting early diagnosis. Limitations might include the limited availability of these techniques in some centers worldwide.

#### Molecular imaging

^123^Iodine-MIBG myocardial scintigraphy reflects post-ganglionic sympathetic cardiac innervation, which is usually reduced in DLB[56], and has similar sensitivity and specificity to DAT imaging [60]. It’s accuracy in identifying LB disorders varies depending on time of acquisition, with the early heart-to-mediastinum (H/M) ratio having a sensitivity and specificity of 70% and 96.2%, respectively; while delayed H/M ratio a sensitivity and specificity of 80% and 92.3%, respectively [56, 61]. Komatsu et al. (2018)[62] showed after 3 years follow-up, abnormal ^123^Iodine-MIBG uptake has a sensitivity of 77% but a high specificity of 97% in differentiating DLB from AD patients, confirming the diagnostic usefulness in early stage of DLB [62].

Moreover, in DLB patients, the pathologic basis and the differential diagnostic performance of amyloid-PET are not (yet well) established, although, it has been shown that amyloid positivity is present in 51% of DLB cases [63]. Different plaque characteristics, including size and density of Aβ fibrils, influence amyloid-PET scan signals and, consequently, DLB diagnosis and management [64]. Kantarci et al. (2020)[65], in a cohort of 39 subject diagnosed as probable DLB ante-mortem, or confirmed autopsy LBD, found that lower global cortical [^11^C]PiB standardized uptake value ratio (SUVr) distinguished cases with LBD from cases with AD or mixed pathology with an accuracy of 93%, concluding that the presence of extensive diffuse Aβ pathology majorly contributes to elevated [^11^C]PiB uptake in LBD [65]. In a recent mixed memory clinical cohort study, using the Centiloid (CL) quantification method[66] for quantifying amyloid burden detected with [^18^F]Florbetaben, AD patients showing the highest amyloid burden, followed by DLB patients, with a mean CL burden of 64.1 (± 26.5) for AD and 49.9 (± 35.9) for DLB [67].

To date the relevance of amyloid markers for the differential diagnosis of DLB from AD is thus limited, however converging evidence suggests that they could play a key role in capturing DLB heterogeneity and possibly as prognostic markers. The assessment of Aβ accumulation, its quantification and its relationship with disease progression in DLB is becoming more relevant, especially from a phenotyping characterization standpoint.

Among the advantages of these methods is important to note: the possibility of identifying AD-related co-pathologies and their help in clinical phenotyping. Their high costs and limited access might represent a limitation.

### Fluid biomarkers

Fluid biomarkers provide a less invasive path for measuring central disease processes in DLB, which will help in diagnosis, disease monitoring, and stratifying patients for trials [68]. Although CSF measurement has traditionally been the gold standard method for the detection of neurodegenerative disease biomarker, blood-based biomarkers are progressively well-positioned because they are extremely easily accessed and scalable in a clinical environment.

#### CSF biomarkers

##### α-synuclein Seed Amplification Assays (SAA)

Α-synuclein seed amplification assays (SAAs) are well-established biomarkers of synucleinopathy. Ongoing research is exploring if the assay can detect patient heterogeneity and allows for early identification of at-risk groups. For example, several studies have shown that SAA analysis of CSF have great sensitivity and specificity (90%−100%) in identifying iRBD subjects, regardless of the future phenoconversion diagnosis [37, 69, 70]. Real-time quaking-induced conversion (RT-QuIC) analysis of CSF also showed high sensitivity and specificity (both 90%) in identifying iRBD subjects, and α-synuclein positivity has been associated with higher risk of subsequent phenoconversion to Parkinson's disease or dementia with Lewy bodies [71]. A landmark multicenter study by Quadalti et al. (2023) [72] evaluated the RT-QuIC assay in 874 patients with neurodegenerative dementia. Among these patients, CSF SAA identified LBD cases with 95% sensitivity and 98% specificity, including those with concurrent Alzheimer's pathology. The study demonstrated that CSF α-syn RT-QuIC maintained high accuracy even in atypical clinical presentations, reinforcing its value as a core diagnostic marker. Importantly, its performance was consistent across various disease stages, including prodromal DLB and mild cognitive impairment with Lewy bodies (MCI-LB) [72].

These results are of particular relevance for patient selection in neuroprotective trials, especially when evaluating therapies targeting α-synuclein. The utility of these methods lay on their importance in staging and stratification of patients to be included in clinical trials, although the lumbar puncture represents an invasive techniques.

### Classical AD Markers (Aβ and Tau)

CSF Aβ_42_ levels are typically reduced in DLB patients with AD co-pathology. Elevated total tau (t-tau) and phosphorylated tau (p-tau) levels indicate tau pathology and are associated with worse cognitive function and lower expression of DLB core features like parkinsonism and RBD.

Ferreira et al. (2020)[73], in a large cohort of DLB patients (n = 417), have tested whether Aβ and Tau positivity, assessed as CSF amyloid-β _1–42_ and phosphorylated tau, would determine a specific clinical DLB phenotype. Thirty-nine (39) % of the sample was classified as A-T-, 32% as A + T-, 15% as A + T +, and 13% as A-T +. A + and T + increased with age in both women and men, with the former increasing more in APOE ε4 carriers with age. Moreover, in the A + T + group, they observed a lower global cognitive performance. On the other hand, Tau pathology was associated with a lower frequency of DLB clinical features, such as parkinsonism and probable RBD [73].

#### Blood biomarkers

##### Phosphorylated Tau (p-tau181, p-tau217)

Advances in technology and methodology currently allow the identification of pathology-specific biomarkers in blood plasma, potentially leading to development of blood biomarkers for AD pathology within the LBD spectrum.

However, little is still known about plasma biomarkers in LBDs. P-tau181 and P-tau 217 have been proven to enhance the identification of LBD patients with and without evidence of AD co-pathology as measured by CSF Aβ or PET tau [74]. P-tau181 has been shown to progressively increase with age in LBD cases, while is found high early on in the AD course[75]; as well as was able to best identified amyloid and tau PET abnormalities in DLB patients, supporting peripheral biomarker detection of AD co-pathology in Lewy body disease [76].

It remains unclear whether plasma biomarkers have sufficient sensitivity and specificity in differentiating between AD, LBD and co-pathology cases.

The main benefit of blood-based biomarker is their minimal invasiveness and their usefulness in longitudinal monitoring.

## Other

A multicentric study involving a clinical cohort of PET-Aβ-positive MCI, AD and positive DAT scan or MIBG DLB subjects showed that higher levels of GFAP were found in both AD and DLB patients, p-tau181 modestly discriminated MCI + AD cases from DLB, while Αβ42/40, Neurofilament Light Chain (NfL) and Glial Fibrillary Acidic Protein (GFAP) poorly classified the same cohorts [64]. Diaz-Galvan et al. (2024) investigated plasma markers across the clinical spectrum of LBD, including iRBD, MCI-LB and DLB patients, and they found that GFAP was elevated early (MCI‑LB) and correlated with amyloid PET positivity, while NfL rose significantly in full-blown DLB [76]. In a Japanese prodromal cohort (NaT-PROBE), NfL increases were evident even in early, high-risk subjects without overt AD pathology, suggesting that axonal damage reflects early synuclein-related degeneration. This finding strengthens the value of NfL as a progression marker in synucleinopathies, including DLB and PD [77].

When comparing PET-Aβ-positive with PET-Aβ-negative LBD, no differences were found in the distribution of biomarkers, suggesting little ability of plasma biomarkers to identify concurrent AD pathology, particularly concerning brain amyloid levels [64]. Although, Vrillon et al. (2024) assessed a broader panel of markers, accounting also for inflammation in DLB patients. They found that, in DLB patients, p-tau181 reliably identified coexisting AD pathology, while elevated YKL-40 pointed to inflammatory processes as contributors to DLB heterogeneity. This underscores the utility of combining neurodegeneration, amyloid, tau, and inflammation markers for disease staging [78].

Considering the low cost, reproducibility and accessibility of plasma biomarkers, further research in the field is warranted to realize robust results that will enable their assimilation into clinical practice.

Key Fluid Biomarkers in DLB and their diagnostic utility, along with relevant studies are summarised in Table 2 and Table 3.

**Table 2:** Key Fluid Biomarkers in DLB and Their Diagnostic Utility

Biomarker	Sample Type	Pathology Targeted	Clinical Utility
α-syn RT-QuIC	CSF	α-synuclein aggregates	Highly specific and sensitive for synucleinopathies
Aβ42/Aβ42/40 ratio	CSF, Plasma	Amyloid-β deposition	Indicates AD co-pathology in DLB
p-tau181/p-tau217	CSF, Plasma	Tau pathology	Identifies AD-like changes; less elevated in DLB
Total tau (t-tau)	CSF	Neuronal damage	Elevated in AD and some DLB cases
Neurofilament light (NfL)	CSF, Plasma	Axonal degeneration	Non-specific marker of neurodegeneration
GFAP	Plasma	Astrocytosis	Elevated in AD and DLB; less discriminative alone

**Table 3:** Summary of Major Studies on Fluid Biomarkers in DLB

Study	Biomarkers Studied	Findings
Ferreira et al. (2020)	CSF Aβ42, p-tau	A +/T + profile linked with worse cognition and fewer core DLB features
Rossi et al. (2021)	CSF α-syn RT-QuIC	Sensitivity: 95%; Specificity: 97% for prodromal DLB
Hall et al. (2021)	Plasma p-tau181	Identifies AD co-pathology in DLB; rises later compared to AD
Iranzo et al. (2021)	CSF α-syn RT-QuIC in iRBD	Positive assays predict conversion to PD/DLB within longitudinal follow-up
Bellomo et al. (2022)	CSF α-syn SAA	High sensitivity/specificity across synucleinopathies; useful in iRBD
Chouliaras et al. (2022)	Plasma GFAP, p-tau, NfL	GFAP elevated in both AD and DLB; plasma markers show overlap
O’Brien et al. (2022)	Plasma Aβ, tau, amyloid PET	Aβ pathology frequent in DLB and associated with accelerated clinical progression
Diaz-Galvan et al. (2024)	Plasma GFAP, p-tau-181, NfL, amyloid/tau PET	GFAP elevated early (MCI-LB); p-tau-181 and NfL increase later. p-tau-181 best predicted amyloid/tau PET positivity
Vrillon et al. (2024)	Plasma Aβ42/40, p-tau-181, NfL, YKL-40	Alterations in all markers observed in DLB. p-tau-181 indicated coexisting AD pathology
Hiraga et al. (2024)	Plasma Aβ composite, p-tau-181, NfL, α-synuclein	NfL elevated in prodromal LBD before AD pathology appears; reflects early synuclein-related degeneration

### Neurophysiological biomarkers

In addition to imaging and fluid-based methods, neurophysiological biomarkers, specifically electroencephalography (EEG), have been valuable tools in the early diagnosis and characterization of DLB. Such modalities are highly promising considering that they are non-invasive, inexpensive, and have the potential to show real-time cortical dysfunction.

Subsequent studies, such as the extensive meta-analysis of Donaghy et al. (2023)[79], highlighted the promising diagnostic role of EEG in prodromal and early DLB. In the current study, EEG slowing in posterior areas was uniformly related to both the presence of visual hallucinations and to impaired cognitive function. These results are of particular significance for MCI-LB individuals, for whom visual hallucinations are an early and discriminating symptom.

The authors propose that these EEG findings reflect a "top-down" pattern of disease with initial cortical involvement dictating clinical symptoms. In contrast, imaging biomarkers such as [^123^I]FP-CIT SPECT (DAT imaging) and ^123^Iodine-MIBG myocardial scintigraphy will more readily identify cases with RBD and parkinsonism, suggesting a "bottom-up" progression of the disease beginning in subcortical or peripheral autonomic structures.

## Conclusion

In conclusion, the heterogeneity of dementia with Lewy bodies presents significant challenges for diagnosis, treatment, and management. The diverse clinical presentations, varying neuropathological progression, and the influence of co-pathologies highlight the need for a more refined understanding of this condition. This complexity underscores the importance of shifting toward a biological rather than purely clinical definition of the disease, facilitating more precise diagnostic and therapeutic approaches. Novel approaches to neuroimaging and genetic research, especially with *APOE* ε4 and *GBA* mutations, offer promising avenues for identifying distinct subtypes of DLB and their disease trajectories. Further research into the pathophysiological mechanisms and biomarkers of DLB will be critical to developing personalized treatments that address the specific needs of patients, ultimately improving outcomes and quality of life.
